# Experiences of citizenship in precarization: An ethnography from northern Colombia in the time of COVID-19

**DOI:** 10.1016/j.heliyon.2021.e07659

**Published:** 2021-07-27

**Authors:** Wilhelm Londoño Díaz, Anghie Prado Mejía

**Affiliations:** a Universidad del Magdalena, Santa Marta, Colombia; b Universidad del Cauca, Popayán, Colombia

**Keywords:** Pandemic, Poverty, Environmental crisis, Immigration, Caribbean, South America, Colombia, Fishermen, Subsistence, Conflict

## Abstract

This article is the result of qualitative research on current ways of being a citizen in northern Colombia during the era of COVID-19. In a region characterized by impoverished communities, the study area becomes a privileged place in which to understand how communities in so-called “developing countries” face a pandemic with their local resources. To place the reader, we discuss the history of this place, the town of Taganga, located in the city of Santa Marta in northern Colombia. Likewise, its culture and social structure are described, as well as the current situation related to access to electricity, water, garbage collection, and food. The article shows how, through local means, today's communities seek to establish settlements with essential services, such as water and electricity, without any relationship with the state.

## 1 Introduction

The current pandemic caused by COVID-19 has highlighted several things. In the Western world, the current models of the nation-state based on a corporatist orientation have undermined the old welfare state, leaving most of the population without medical coverage. Instead of seeing preventive health conditions improve as the global population grows, the nation-state has been privatized so that social investments are reduced. This is not a characteristic of developing countries, and of course there is also poverty within the global North (Kacowicz, 2007). The pandemic has shown that civil society in the Western world is unprotected, especially in Latin America, where right-wing governments thrive that make little investment in maintaining decent living conditions for their populations. In Latin America, the effect of confinement has made evident the limited access to connectivity of a large part of the population and revealed its limited access to health services (Molina Prendes and Mejias Herrera, 2020). With a state that invests little in health, such as those in Latin America (Rodríguez et al., 2020), the statistics are clear: there are problems of malnutrition, low access to education, health, and recreation. The consequences of this are apparent: little development of high-quality human resources in the region, low technological development, and a tendency to produce commodities and few value-added products.

If, quantitatively, the data show a pauperized Latin America (Giraldo, 2015), the corresponding question is: How do current citizens generate living conditions in environments where there is no state aid or assistance? How are new citizenships generated in the peripheries where there are structural conditions for the reproduction of poverty? This is the question that surrounds this research and that it tries to solve in a place with a high rate of vulnerability in its population, given its demographic characteristics. This set of traits can be summarized in a description of the place as a village with a high floating population with little purchasing power.

Given this situation, we propose an analysis of how settlements of people outside of any social assistance are generated. Since the region of Santa Marta, especially Taganga, is currently a destination for many Venezuelan immigrants, this space becomes a useful one in which to apply qualitative methods, such as participant observation, related to spatial analyses, something already widely used in the social sciences (Matthews et al., 2005). The advantage of this type of analysis is that it allows quantitative data showing correlations to take a different route; in this case, the qualitative data help us understand the trend in depth and provide new views on it. If the trend is a constant disassemble of the state, which is verified in subnormal settlements. And given this situation someone can ask: how do these populations manage to overcome this situation? Thus, participating in these people's daily lives give a glimpse of that process of building a place. In addition to participant observation, a life history methodology was designed to understand the current conditions of these populations, as understood from the global situations that determine them and in front of which there are local, often unusual, responses.

A not-insignificant feature of this research is that, as it is understood how Venezuelan immigrants generate neighborhoods in certain areas of Santa Marta, such as Taganga, it is also understood that there is an illegal land market and that the settlements are sometimes built on lots of private or collective property. Thus, this type of research must address illegality issues, limiting our access to specific details of the ethnographic record (Contreras, 2019).

In this case, given the overwhelming amount of data showing precarious conditions, qualitative research allows us to see how, in the face of adverse trends, other dynamics are generated that are accessible only by qualitative means. In this case, we can appreciate place-building processes (Ramsay, 2020), which imply emulating the entire landscape of average or middle-class neighborhoods. In this way, the process this article describes concerns not a given place but the constant construction of that place, which implies social, economic, and political relationships that go beyond that location.

Regarding the literature of these themes, archaeological techniques have been used to study the homeless population (Rooney, 2016; Zimmerman et al., 2010); some emphasis has been placed on their mobility and how they are related to their survival. This is not done in an empty frame but to make these populations visible to enable interventions. Likewise, other studies have emphasized how local people's building construction involves activism (Martin, 2003). We also find necessary research that shows how neighborhood-occupation processes are generated by various migratory waves in cities like New York (Hum, 2014); Despite this, we have few neighborhood constructions cases like northern Colombia. On the other hand, Colombia does have a long tradition of neighborhood studies (Ramírez, 2006), but these have usually focused on the organizational processes that outline how a group of leaders starts negotiations so that the regional government accepts the neighborhood. Despite this, these studies have not considered the construction of the place or solutions to the fundamental problems of access to water, electricity, and the internet. This was due to the prior trust that had been established in that many of the people to whom we spoke; trust, a staple for qualitative research, was already established in these cases (Gable, 2014).

## 2 Methodology

### 2.1 Ethical approval & consent

The information contained in this document was obtained under the ethical standards of anthropology. Testimonies were taken with the consent of the interviewees, and their identity was protected when necessary. We did this research as a member of the Anthropology department of the University of Magdalena.

This research has the consent of the Indigenous Council of Taganga and is endorsed by the Ethics Committee of the Faculty of Humanities of the University of Magdalena.

The village of Taganga has a history of immigration associated with problematic practices, such as sexual exploitation in tourism promotions (Jouen and Zielinski, 2013). Since the local population is minimally related to the fluctuating population, this site was chosen to explore how, given the slowdown in tourism due to the COVID-19 pandemic, the immigrant population of Venezuelans has tried to create a new neighborhood with its own hands. Once we heard about settlement construction, we located some leaders and asked permission to do some open interviews based on the life-history method (Mallimaci and Giménez Béliveau, 2006). The advantage of this method is that it allows us to capture the subjects’ agency and perceive trajectories that are sometimes contrary to the trends that can be perceived from quantitative methods. In this case, although the data show that Taganga is a village with poor primary sanitation conditions (Meisel-Roca et al., 2018), the impoverished population tries to build meanings and citizen spaces that compensate for these deficiencies. We were able to detect more than 60 people with family relationships, and we decided to work with a nuclear family member who recently occupied an abandoned building to make her home. This selection was made in view of the fact that this family is the one that has the most challenges, such as raising a child, finding food, building a house, and living there. Furthermore, this family had no problems answering our questions. It is essential to clarify that it is impossible to obtain precise data on how many people there are and how many settlements are being built since this new neighborhood is built on land that in practice belongs to the state; thus, this settlement is an illegal usufruct of land. Although this is not the topic of this research, it does generate some restrictions on it, such as in the data on the size and educational level of entire migrant population, for example.

Methodologically, the life story begins with questions that describe a timeline: the before, the during, and the now. Therefore, we tried to review where these immigrants came from (the before, for which we auscultate in daily life before immigration). Many of these people were born in the collapse of the Bolivarian revolution, and others have previous memories of that revolution. In addition to the before, we also asked about the motivations for immigration to Santa Marta (the during); most people emphatically pointed out that the cause of immigration was accelerated inflation. Finally, we asked how their settlements were built and why they chose the place they did. Those questions were asked before the construction of the chains of trusts essential to the enography research were established (Gable, 2014).

### 2.2 Analysis tools

This article starts with some necessary theoretical premises. The first is that there are no groups, but rather group formations (Latour, 2008; Sayes, 2014). According to Latour, many actors allow human associations called groups. Thus, ethnographic descriptions should start from the premise not that the group is a consolidated entity but rather that it is something under permanent construction. According to these theoretical postulates, when we create an ethnography, we observe not the group but the simultaneous action of agents that guarantee the homogeneity and construction of the group. As Latour points out, to maintain a certain coherence, groups must occasionally reinforce their members’ loyalties through rituals involving diverse variables like material culture, music, dance, and food. It is evident that, with the advent of modernity, the state would be the institution in charge of generating the references for the groups called nation-states. In this way, contemporary identities are constructed or give form to groups using the nation-state model, but the maintenance of local cultural values also plays a fundamental role.

At present, Taganga is a source of immigration for Venezuelans who come mainly from the Maracaibo region, on the border with Colombia's eastern border. As this diaspora migrates in the tension between the new Cold War between Russia and the United States, these populations enter Colombia without any state support in such a way that they must find with their own means where to form their settlements to live. By observing how individual Venezuelan families begin to form neighborhoods, we can get closer to perceiving contemporary forms of construction of groups of citizens living on the peripheries' fringes, such as in the north of Colombia. Since the settlements made in Taganga are in some sense illegal, it is difficult to determine how many families have arrived in recent years. Nobody wants to talk about this land tenure. One way to solve this methodological problem is to use spatial information about the new occupation areas.

From a methodological point of view, most of the information was collected from a nuclear family of Venezuelans who, for privacy reasons, are not shown in photographs. Still, this family, comprising a woman, man, and young child, exemplify or are paradigmatic in expressing the current needs of these immigrants and how they solve problems, such as access to food, water, electricity, internet, and a sense of citizenship. Since the Colombian government offers only medical assistance to Venezuelans and there are no plans for their nationalization or any educational offerings for them, these populations also provide themselves with national symbols, such as soccer jerseys or local music consumption. Thus, understanding what nationality is and how it is currently negotiated is a crucial point to understand.

In Appadurai (1996; 2001) case studies of India, the processes of national identity formation took place on a substrate of connections that had generated the mass sports that arrived with colonization, such as cricket (Fletcher, 2011). In this way, Gandhi's political movement made a call on an imagined community that appealed to local religious values through a communication network established by the British. In this way, the identities of the newly formed states that achieved decolonization in the twentieth century were found through their relationships with radio, press, and television, and external elements, through which they mobilized local images based on religious dogmas. As Geertz (1973) shows, in Indonesia's case, the political parties called for the formation of a nation, either by appealing to the adoption of modernity and its promises of freedom or to what was considered the legacy of tradition. We know that Geertz called these contents *ideology* and did not explore an understanding of how these contents were created, circulated, or consumed in socio-environmental and technological networks (Throop, 2009). We know that this deficit was overcome by the theory professed by Latour (2008), who drew attention to the understanding of human groups with a constant construction that involves human and non-human actors. Given the role of non-humans in the formation of social action, Latour has called his approach Actor–Network–Theory (ANT).

These ideas are relevant because the description we will provide shows how groups are currently being generated and how they create local responses to the COVID-19 pandemic situation. In this way, ethnography would not be oriented toward describing a specific type of group but rather how groupings are generated in the spaces of the global South characterized by the pauperization of society. Although an analysis of what pauperization means in the Anthropocene era (an era characterized by a global crises) is understandable, we must try to make ourselves an image of what it means to build citizenship, being a pauperized citizen, in the middle of a pandemic.

Returning to our theme, we can say that, although human beings, from the biological point of view, are equal, it is evident that group construction processes occur in diverse ways and at diverse scales around the globe. For example, the identity-building process of a Nordic nation where there is an absolute consensus about the past, is not the same as that of the Zapatista movement in Mexico, which is struggling to decolonize the representation of indigenous groups that is the foundation of Latin America (Saldaña-Portillo, 2002). Both processes, however, share the use of non-human agents to construct the networks that give them a sense of homogeneity. As stated, these agents allow the development of what Anderson (2006) called “imagined communities”; at any rate, we must understand that Anderson's theorization was gestated to understand the processes of identity formation in the nation-state era.

In sum, it becomes clear that imagined communities are groupings based on content called *ideology* that is blurred by various networks involving various scales of technology and material culture. Likewise, these groupings take multiple forms depending on their colonial or postcolonial contexts. Nation-building processes in the global South and the Atlantic North differ. The dilemmas of feeling Colombian in the north of Colombia are not the same as feeling Danish or Swedish in Catalonia or Paris.

The case I will present is that of a group of people currently occupying the upper parts of Taganga Bay. According to preliminary investigations and interviews, these are Venezuelan migrant families who arrived in the region in recent years (no more than four years ago) and who, faced with the mandatory quarantine, decided to stay in the city, resulting in their having to face the problem of obtaining housing that met minimum conditions, such as a roof, electricity, and water. The ethnographic work done using participant observation allowed us to understand how the housing site's formation has been taking place and how these new homes are being articulated to the existing networks of electricity, water, and social ties.

In view of the area's illegal occupation, it was not possible to collect data on how many families there are and how much of the area has been occupied. A satellite image, however, was used to see the occupation pattern of the area. Four new houses built in the pandemic between March and May 2020 were selected from the highest part of the bay, and the research proposal was presented to their occupants. Of these four families, one agreed to provide information if it was given a guarantee of anonymity.

**Figure 1:**
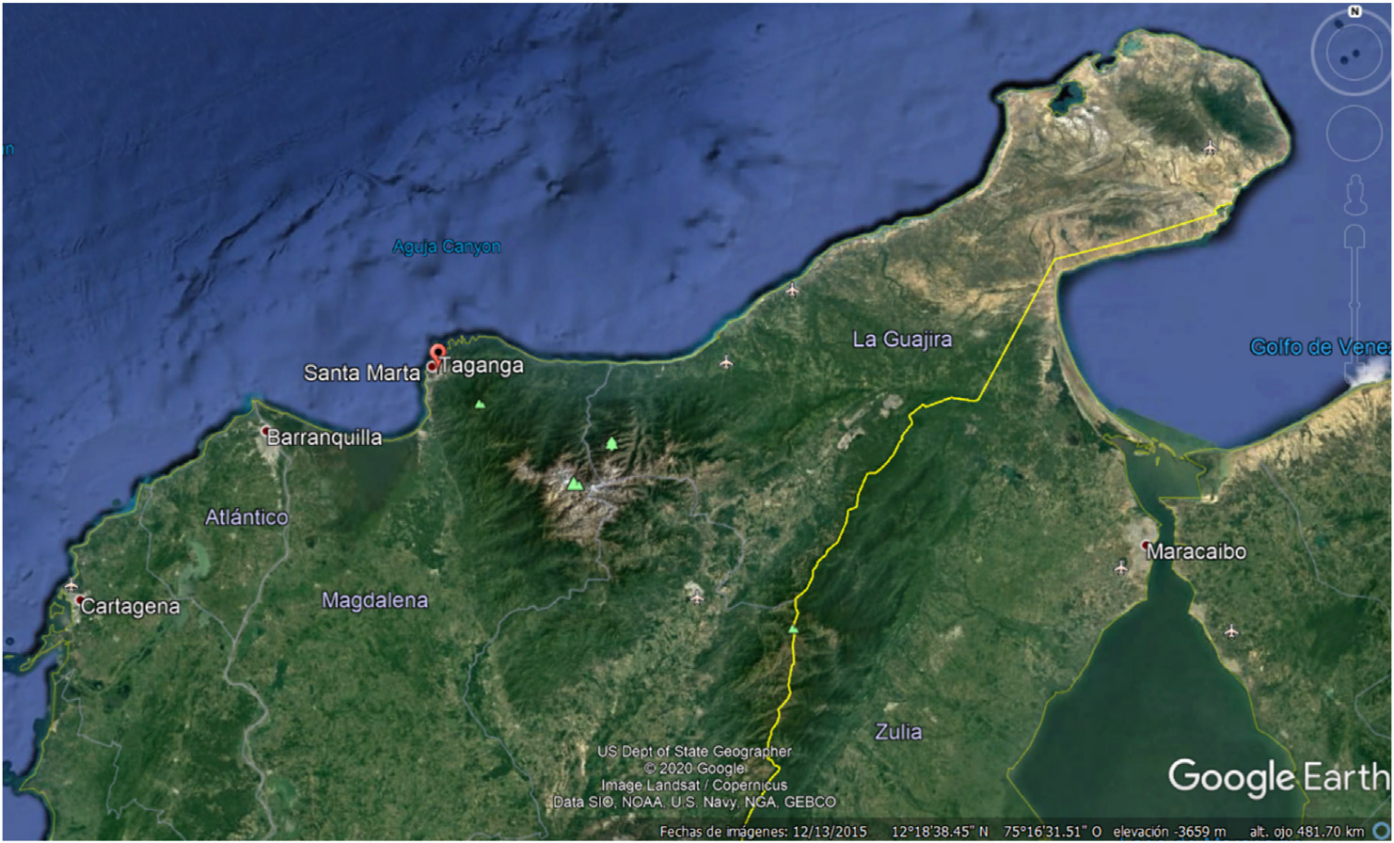
Map of Taganga.

The work area, which not cover one square kilometer (see Figure 1), currently has approximately five families settling there, which have built almost two dozen new houses. Similarly, the people we spoke to noted that the settlements are legal; at first glance, however, the work being done does not comply with official standards, such as one that the workers must have biosafety protocols in place to prevent the spread of COVID-19, nor are the regulatory signs indicating the start of work.

The families generating these new settlements are mostly from the Maracaibo region of Venezuela. They are primarily young people who came during the last wave of immigration that took place in the two years before the pandemic. The people generating these new neighborhoods have no technical or professional training and migrated to Colombia because inflation made the acquisition of minimum goods unsustainable. In addition, in the conversations held with them, it became clear that their place of origin presented high rates of violence, so that Colombia represented an opportunity. In this regard, Pablo, a Venezuelan immigrant in Santa Marta, said:

My son and I wear 12 in American sizes, and that is hard to come by over there; in about 2010, you could still get the money together and buy some pairs ordered from Miami. However, then the prices became unaffordable. It was better to come to Colombia, where it is even easier. (Interview, July 20, 2020)

We must remember that the historiography of Venezuelan immigration to Colombia emphasizes a sort of golden age, where great professionals arrived and transferred knowledge (Hernández, 2011). The last immigrations that Pablo mentions occurred after Hugo Chavez's death and were characterized by the departure of people from lower socioeconomic strata. Thus, these new socio-cultural configurations occur in historical plots that must be clarified.

Consequently, to understand this case, we must understand the space where this resettlement takes place. Therefore, we will describe the place and provide a historical orientation so that the reader can understand the geographical and historical context of Taganga.

Some important notes should be made before we continue. We have been living in Santa Marta for several years and in Taganga for three. We have participated in various internal political processes, such as helping the Colombian state recognize some Taganga families as indigenous. In addition, several local leaders have been our anthropology students at the University of Magdalena, so we have a local recognition that is difficult to hide. This makes the information we get certainly privileged, while it compromises us with the people who live there, as they are our neighbors. Despite this conflict of interest, we will try to show a transmission of the experience of the pandemic from a place in the world that is not the most privileged, nor the most representative of a country, but a complex human condition, which we should make visible to generate the necessary transformations of the current society. Here we should note that Franz Boas (1904), the father of ethnography, pointed out that all anthropological research contributes to the generation of a global conscience of the human condition. That is what we want to do.

### 2.3 The study area

The village of Taganga is located north of the bay of Santa Marta (see Figure 1). The village has been occupied since about 1300 BP; we know this from the archaeological evidence that has been obtained in recent years (Londoño, 2011). Archaeologists do not rule out that human occupations on the coast may be older; for now, the dates of C-14 indicate that. From the archaeological and linguistic data available, we know that the Sierra Nevada de Santa Marta coast was occupied some three millennia ago by populations from Central America, especially Honduras (Constenla, 1995). These groups, from the macro-Chibcha linguistic trunks, would have settled in the savannas adjacent to the mountain range and begun a dispersion through the inter-Andean valleys; the last spaces to be inhabited would be the eastern valleys that form a narrow pass formed by the Sierra Nevada de Santa Marta and the Serranía del Perijá.

**Figure 2:**
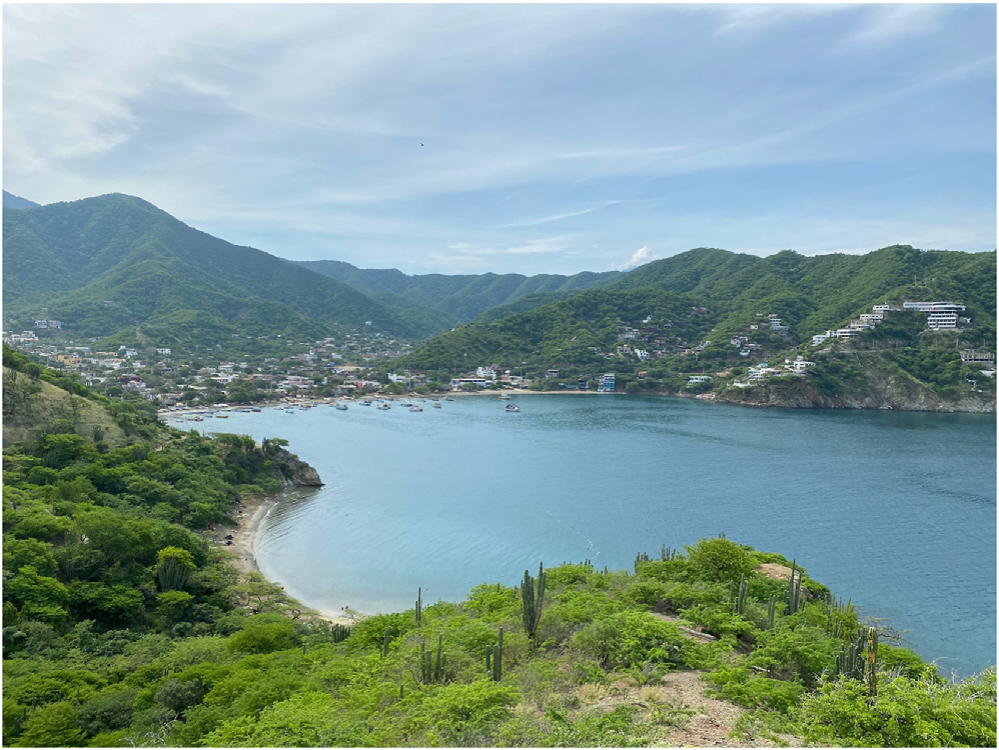
Taganga bay.

Various colonial and early republican documents report the presence of fishers in the village of Taganga, and the crown issued titles certifying ownership of coastal lands from Taganga Bay itself to the north in what is now the Tairona National Natural Park (PNNT; Langebaek Rueda, 2015). Due to environmental conditions, this area is formed by tropical dry forest (Carbonó and García, 2010) with a predominance of xerophytic vegetation. The forest covers some scarps that go directly to the bottom of the sea to form coral environments. In this area, the bays form small beaches with sloping sides, used for fishing with nets called *chinchorros* (see Figure 2). From the information we have available from North American explorers who arrived in the area at the beginning of the twentieth century (Londoño, 2020), we know that, from Taganga, there was maritime traffic to the city of Riohacha in the province of La Guajira, on the border with Venezuela. We also know that there was maritime traffic to Cartagena de Indias, which was done in small sailing boats.

At the end of 2019, the Colombian state recognized that in Taganga there was an indigenous council, so the area, besides being a center of immigrant settlement, is also a site where political processes of ethnogenesis are developed (Arruti, 2000). Likewise, Taganga, before the pandemic, was recognized as one of the main tourist destinations of the region, so it is clear that there are several simultaneous processes managed by various actors with different powers in this place. We should not forget that, years ago, the Colombian government expelled an Israeli pimp who had turned the village into a sex-tourism destination. This space, then, before COVID-19, was a destination for sex tourism, a destination for beach tourism, a place of ethnogenesis, and at the same time, a destination for immigration.

### 2.4 History

According to existing documentation, the village of Taganga was described in the 18th century by a series of civil engineers who began to produce the first plans of Santa Marta's town. Likewise, the village is mentioned in various colonial documents, and there is not necessarily talk of the assignment of an *encomendero*, probably because the Spaniards were not interested in dominating a fishing village; for them, it was more profitable to dominate the mountain villages because there they could tax non-perishable products, such as corn, livestock, and wool.

At the beginning of the nineteenth century, when the republic was founded, Santa Marta was inclined to maintain the monarchy, and since it was a small port, it did not play a crucial role in independence, as Cartagena de Indias did (Calvo-Stevenson and Meisel-Roca, 2011). In any case, neither was a considerable effort made with the republic to control the village of Taganga because it offered nothing that the Colombian state most sought in the Indian reservations: men for the civil wars (Londoño, 2020). In the twentieth century, after the Second World War, the village became destination for a particular type of tourism: young Americans traveling to the Colombian Caribbean in search of narcotics. Although this tourism did not create the marijuana business, which was already operating even for a small domestic market (Osorio, 2018), what did happen was that a damning regulation of the production and consumption of this plant emerged. These prohibitions generated a polarization in which the south became the supplier and the north the consumer; parallel to this, the anti-drug policy was consolidated by targeting the producers in the south but not the distributors in the north, where the real wealth in drug trafficking is generated (Tokatlian, 2008).

According to an interview we conducted with an older man who participated in the marijuana bonanza (whose name we will conceal for legal purposes):

I grew up in Pescaito, the neighborhood of soccer player Pibe Valderrama. We used to go to the beach to fish, and when I grew up, they gave me a motorboat that we used to fish around Punta Aguja. Then some guys from Dibulla came and offered us fifty thousand pesos from that time to take a load of Bonda to Chengue. I worked with them for about two years, and we drank and went whoring. Then I saw that I was running out of money and I bought a lot here, and a small lot through the SENA agricultural service. (Interview, March 12, 2020, in Taganga)

In the 1970s, the demand for marijuana and cocaine coming from Florida allowed for an operational chain that generated wealth along its length. Many young people from Santa Marta began working for “gringos” who came with suitcases full of dollars ready to buy cocaine or marijuana. Some young people simply drove small boats with the mission of taking the merchandise from the coast to the speedboats waiting on the high seas. Other young people, working on the plantations or in the cocaine laboratories, as well as many others, began to work in the hotels and restaurants that emerged after capital began circulating in the region. The hotel industry was one way in which drug-trafficking money attempted to be invested locally. Once the marijuana business began to decline, businesses like hotels or restaurants, which lived off that bonanza, began to decline. Even today some ruins from those times can be seen in Santa Marta.

If there is one defining characteristic of the second half of the twentieth century for the region of Santa Marta, it would be a land market in a moment when the area carried the image of a lost paradise. Another of its distinctive features was the re-ordering of the territory under conservation policies (Serje, 2008). In this way, the region began to be contested by state bodies and parastatal agents, which ended up generating pressure on local communities. With the opening of the land market, the privatization of previously uncultivated areas began. In fact, a review of the documents at the Office of Registry of Public Instruments in Santa Marta reveals that most properties were titled as of 1999; in that year, the territorial entity, the Santa Marta mayor's office, gave lots to local private individuals, who then resold them to businessmen (see file Registration No. 080-95653).

With the privatization of the land in that decade, the people of Taganga lost their dry forest areas, where they usually went to cgvut wood for their daily tasks. With the privatization of these areas, the local people lost autonomy, and their territorial control was reduced, decimating local governments’ agency capacity.

One of the most significant effects of this territorial reorganization was capital investment in the real state. In this way, Santa Marta's surroundings, including Taganga, began to undergo a rapid land privatization process. Many drug traffickers invested their initial fortunes in tourism-oriented real estate and lots that they used to retain and increase capital. This is how the El Rodadero resort was built, which retains the architecture of the 1970s (Herrera, 2012).

In the specific case of Taganga, the 1970s involved a land-titling process that meant that many natives gave up their properties. As a result, the higher parts of the village became private property. From that decade on (1970), construction of country houses began to accelerate in the early 2000s. In this sense, the last two decades have been the scene of a real-estate boom that can best be translated as a *deterritorialization* (Vilas, 2004).

The real estate pressure created a stratification of the land; people gave up their lots in the upper areas to external buyers, both national and foreign, and started to inhabit small enclosures in the lower regions. The local people now live in the lower parts in houses made of concrete blocks with zinc roofs, on lots that do not exceed 70 square meters. By contrast, non-local owners have large extensions in the higher areas in the hills leading to Bonito Gordo and Bahía Concha's beaches.

**Figure 3:**
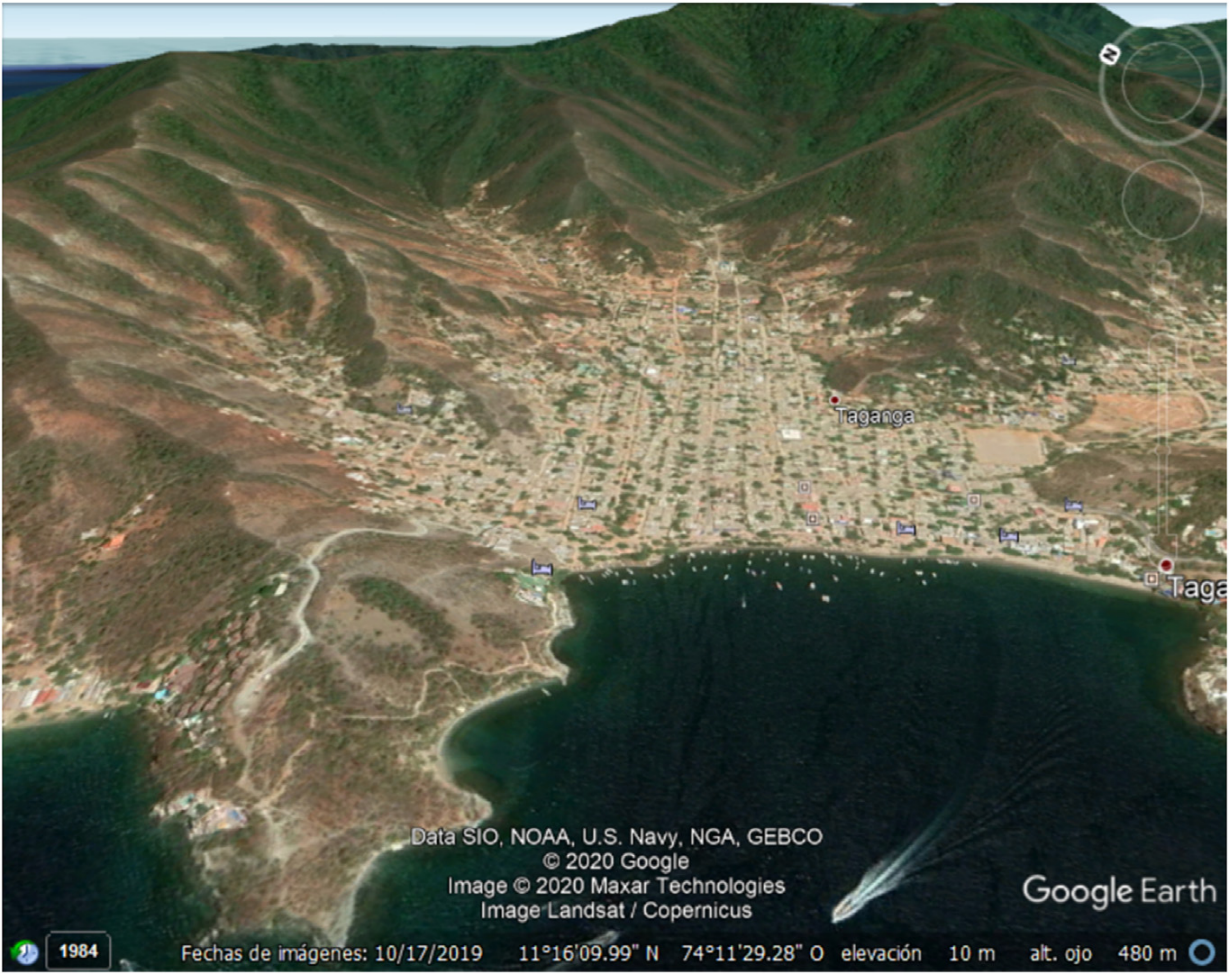
Land-privatization map.

As seen in Figure 3, taken from Google Earth, the lots are more extensive in the higher areas. This is the spatial expression of the land-privatization processes mentioned above. When we examine the real-estate registrations of some properties, we see that the houses for these sectors were built a couple of decades ago. Currently, above that belt of select properties, a process of reoccupation is taking place, this time not by people with high purchasing power but by some nuclear families of Venezuelan origin who did not escape the quarantine that began in March 2020 and ends in a September 2020.

These families, who will be discussed later, occupy lots that are currently being used to dump debris from other buildings illegally. In other words, these families live in waste areas with no connection to electricity or drinking water. The occupation of these areas took place in the dry forest that was for practical purposes the communal territory of the Taganga natives. These were the areas that failed to be bought by the large landowners who expropriated the natives of the bay in the first wave of property sales.

Since there is no drinking water in the village, water management and access to water is an expression of the inequalities of the Taganga people, as well as an expression of social formations that grow without a supply of drinking water (Rockström et al., 2014). To compensate for these water deficiencies, owners of country houses buy drinking water from suppliers in Santa Marta, while immigrants and the precarious population are supplied with water from a tanker that comes by every week. People are obligated to be very strict with their water consumption, as no more than 15 L per day are available per family. These data were obtained by participating in the filling of containers with capacities of up to 5 gallons. Each family is allowed up to three of these plastic containers, called *pimpinas*.

### 2.5 The culture

At the end of 2019, the Colombian state recognized that, in Taganga, there was an indigenous form of government; this recognition granted them legal instruments that accredit them as indigenous people. This recognition was given to the families who wished to participate in the process, which formally began in 2010, after decades of complaints because the central government was reluctant to grant these ethnic recognitions. The reasons for the refusal are diverse, but among them may be that a specific business sector feared that generating more ethnic autonomy would be required in any future authorizations for the development of civil works in the ports and tourism infrastructure (Vega, 2014).

At the beginning of the twentieth century, Taganga was a fishing village that made logboats from giant trees from the rainforest of what would become Natural Tayrona Park. Their work was essentially divided into two large areas. One was the fishing of *chinchorro*, a mesh that extends along the small bays between the hills and is explicitly used to capture small fishes. The other is open-water fishing performed with harpoons and hooks. Fishery still occupies a large part of the activity of the people of Taganga, and, to the economic and political processes that took place from the 1960s onwards was added an additional layer of tourism. With the advent of tourism, the village became a tourist destination, and soon many people, both Colombian nationals and foreigners, began to settle in Taganga, establishing matrimonial alliances with its residents; others simply settled there to establish tourism-related enterprises. Likewise, Taganga and its surroundings focused on the reception of those displaced by the internal wars of the 1990s. These populations added another layer of population to a village substrate that had already been altered by the demographic pressure of the 1970s.

Taganga has become an exciting site for investigating contemporary immigration. We can appreciate that the town is a recipient of people displaced by violence, as well as Venezuelan immigrants. One main reasons for the desirability of this destination is the offer of impoverished populations resources. On one hand is tourism; in the town it is easy to find employment in various sectors of the tourism business. On the other hand, the sea offers protein that can be accessed easily. Consequently, the site offers living conditions that are not found elsewhere, such as in large cities.

When we examine the village's history in the last third of the twentieth century, we see a growing process of deterritorialization in which villagers were dispossessed of their land by valorizing their territories and the pressure to sell them. On the other hand, we add the layer of those displaced by the violence of the last decade of the twentieth century in Colombia. Finally, we add the layer of Venezuelan immigration over the previous five years. This makes this village diverse: it is possible to see large private mansions, large hostels for backpackers, and small houses with impoverished people from various backgrounds. About this situation, the Indigenous Governor Ariel Daniel points out:

Taganga has always carried a stigma, Taganga has always been looked down upon. A few years ago, the garbage from Santa Marta was coming to our beaches. Then they wanted to put the underwater garbage pipe on our beaches. (Personal communication, April 12, 2020)

**Figure 4:**
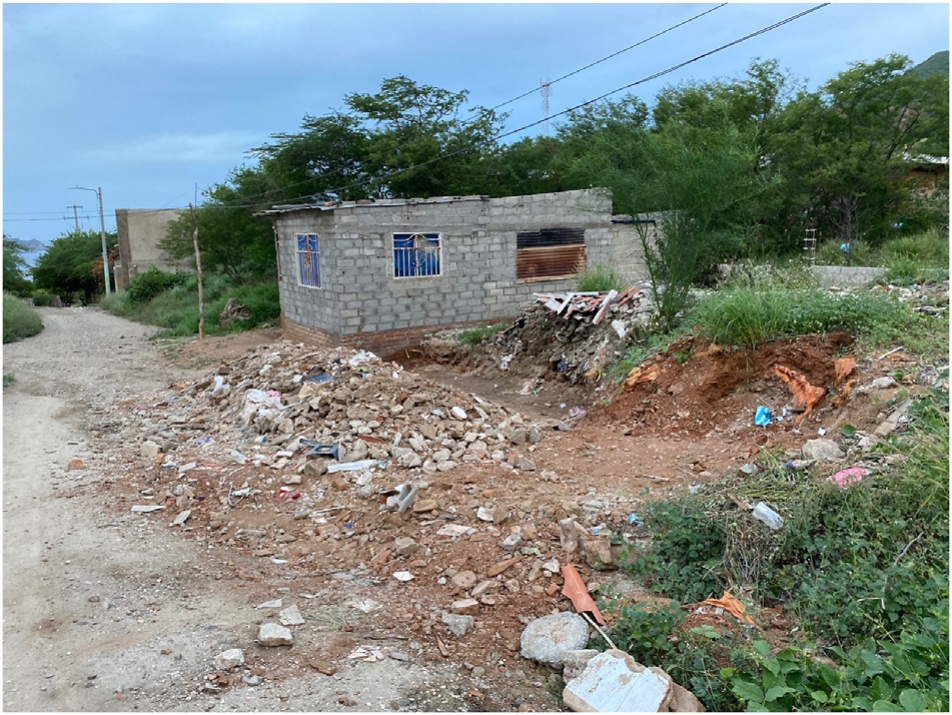
New settlement in Taganga.

Various factors have caused Venezuelan immigration to Taganga, and people from diverse social and cultural backgrounds have migrated there. Obviously, those who have come to build settlements in precarious conditions have done so because returning was not an available option. Likewise, these populations have decided to populate the last flat parts of the dry forest after the country houses. It should be mentioned that behind these cottages is a longstanding area for garbage disposal. As a result, these last inhabitants have begun to build their enclosures on small lots that have some rubble. Consequently, the dry forest that initially provided wood to Taganga's natives has become a garbage dump for the country's builders, and on this rubble, the Venezuelan families have begun to build their settlements (See Figure 4).

These new inhabitants have had two options for establishing up their settlements; they have reoccupied small existing structures that had been abandoned by their owners, usually people with a high level of itinerancy throughout South America, by hitchhiking and selling handicrafts; on the other hand, they have also begun new construction in spaces considered vacant or whose ownership is not very clear. In Figure 4 we can see a family's settlement: a man and a young woman who in April 2020 had three months pregnant. Next to the enclosure, another person is building a terrace to raise a structure whose walls are cement blocks and roofing it with zinc or aluminum cans.

In addition to having to solve the problems of the structure of the enclosure, walls, and ceilings, the issues of connectivity to electrical networks, drinking water networks, and sewerage networks must be solved, to say nothing of the networks responsible for the flows of protein, carbohydrates, and calories needed to live. These sectors, once a virgin dry forest, have no connectivity of this kind, and it is only through self-management that these settlements have begun to meet minimum conditions of habitability in a scenario where there is neither supervision nor cooperation accompaniment from government agencies.

Since this research implies a good understanding of the forms of land appropriation by immigrants, much information must be kept anonymous because specific names and places could compromise these populations’ actions. In any case, the accounts obtained, which recreate the controversy over these new settlements, indicate that a sector of the Taganga population, including natives, have authorized the immigrants to build behind the rubble in exchange for cash. The sums we were able to identify range from $200 to $300, considerable sums if we consider that these people would earn from their daily work, in times of quarantine, perhaps $3 or $4 a day. Before the pandemic, these amounts could oscillate between $10 and $15 from tourist activity. These populations, however, are linked to fishing, so protein should not be in short supply.

After having consulted the Registry Office of Public Instruments of Santa Marta, it seems that these areas do not have real estate registrations. Based on traditional surveys, some local people have given away lots no larger than 60 or 70 square meters. Some sales websites, such as mercadolibre.com.co or olx.com.co, reveal that some of these lots, not only in Taganga but also in the surrounding hills, are sold for up to $3000. Investors in these lots, however, know that they risk being evicted, either by more powerful groups or by local government institutions.

In this respect, a motorcycle dealer who usually invests in these projects and whom we know through our shared love of motorcycles says:

One can go to the beach and make a fence out of a lot. If the police come to take you out, you file a complaint, and the state has to review your case. The process can take up to 10 years, and in those 10 years, you have a lot on the beach. If you throw a ticket to the judge hearing the case, the state must legalize your lot. In the worst case, if you make the fence, a paramilitary can come and threaten you: either remove the wall or give money. Then it's a profitable business. (Interview, 2012)

Here we must highlight, as this person noted, that these territories are dominated by multiple layers of power in a network of tensions that sometimes unleashes violence. In any case, it is possible to observe that there are areas under the full control of the Colombian state, such as the port of Santa Marta and the private ports for the export of coal to the south of the city. The same is not the case in the mountains, where control is exercised by criminal gangs that are the heirs to previous paramilitary groups. On this note, this person argues that, if he buys land without legal documents, he will probably have to ask permission from one of these armed actors who act as parastates.

The appropriation of wasteland has a long history in Colombia, and these processes of land redistribution have never been equitable, but rather have been determined by the whims of local elites. Even in the nineteenth century, indigenous land redistribution methods were attempted, which failed because certain elites prevented it in various ways: assassinating the new owners or swindling them by making them sign promises of sales in exchange for alcohol or tobacco (Londoño, 2003).

If we examine the population layers in Taganga, we see that the space expresses these social differences, which are the product of political practices related to local and global forces. In this space, there is an interaction between layers of legal inhabitants, who connect to electricity, gas, and internet networks—which are the only existing networks because drinking water and sewage networks are non-existent. This means that people may or may not be connected to formal networks, depending on their purchasing power. If they do not have this connection capacity, they must solve these problems by employing popular technologies (Llinas and Dueñas, 2014). These technologies are local strategies through which citizens obtain water and electricity by their own means, connecting to formal networks in non-formal ways. While these dynamics are considered fraudulent or criminal, they are in practice tolerated because the state cannot guarantee the necessary supply to these populations. In Taganga, it is impossible to connect to the drinking water networks because they do not exist, but in the lower area near the lane, the settled people have managed to tap into the primary drinking water pipe.

**Figure 5:**
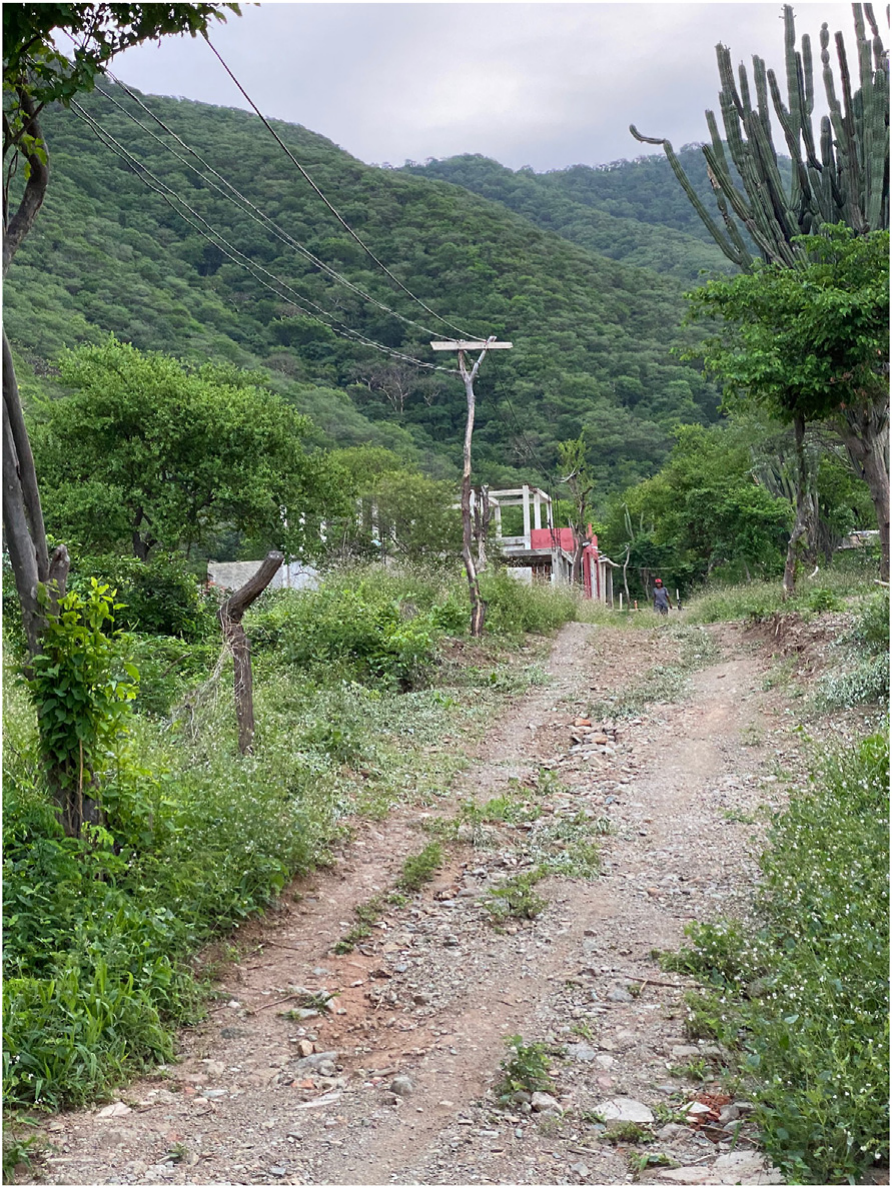
Local electric pole.

As seen in Figure 5, the new settlers create their electric power poles with trees that are pulled from the forest; on these poles they place a crossbar, on which rest the cables needed to power their homes. Usually, this is done to connect residents to the satellite television network of the only provider, DIRECT TV. Since these residents are not in the formal market, they do not have plans with this company, but the company does offer a prepaid service.

From the above description, it is evident that, before the pandemic, a large number of people came to Taganga via irregular settlement processes and lived in precarious conditions; when the pandemic began in these populations, it generated other challenges that taught us that we were in the midst of a crisis, making evident the more profound crises related to the demographic explosion being experienced in northern South America that involves Venezuelan immigrants.

Seeing these realities raises questions: How does one survive during the COVID-19 pandemic in spaces without water, without electricity, and without education systems? And, equally: How do local governments address the humanitarian crises that will be exacerbated in the future? How is the climate crisis being dealt with in the territories of the global South?

We will return to these questions later, but their answers are beginning to emerge. In the current conditions of our planetary existence, where migration is a condition of human reality, millions of people dedicate a large part of their lives simply to solving problems of water, carbohydrates, protein, shelter, and access to television and the internet. This is not an anomaly but a condition of the existence of what some call the Anthropocene. Never before in human history have millions of human beings struggled just to survive on an overpopulated and polluted planet. The problems of immigration are solved in spaces like Taganga, which are not regulated by the state because the state is corrupted by elites focused on receiving the royalties from multinational corporations that legislate for their benefit. This is the drama of the banana republics, enclave spaces where the population is not the priority because the population makes up part of the resources that must be made available to generate capital through the export of commodities; in Colombia, the only exception to the enclave model is cocaine trafficking, which is the only value-added product that is exported, albeit illegally.

Thus, these population strata must solve their problems of settlement generation and articulation to networks of work, education, and essential services, such as water and electricity. As anthropologists, we have always been struck by the fact that, in these settlements, a form of city is recreated, with its informal water and electricity connections, which emulates middle-class neighborhoods. There is a desire by the local people to live by the standards of the middle-class model, and they start building their settlements under this model, even if they are outside social-care networks.

Santa Marta is about to turn 500 years old, and still, the problems of environmental collapse are not being addressed. The pandemic has shown the reality of a city that depends on tourism for its daily life. It is estimated that nearly 300,000 tourists enter the city in December and January, providing resources to an informal population that remains uncalculated but is estimated at around 100,000 people. The arrival of the pandemic cut off these supplies, and the inhabitants stopped keeping cash. The food supply arrived using vouchers distributed by the departmental and district governments, and this aid has been complemented by the family networks that, in the case of Taganga, give Venezuelan immigrants access to fish.

The pandemic shows that tourism in the medium term is not an option and that tourism that has been working only to give crumbs to local people should not be the only way to exploit this commercial vector. In any case, local authorities have not called for a review of how to generate a reorganization of the territory that plays with two variables: the demographic crisis and the climate crisis. In the medium term, local governments will have to face the pandemic's mess. Currently, it is only providing shelter and food to homeless people, which is considered a temporary measure.

### 2.6 The present

As seen, at present, the village of Taganga has a social stratification expressed in its space. The beach line has been occupied by various people who have established small pantries to sell liquor and snacks to tourists. The families formerly settled there were displaced above the beach line. They were also left below the new owners who built their country houses in the foothills. Thus, the upper sections are owned by non-Taganga people who have purchasing power, and the lower parts are owned by local people who share space with internal immigrants who arrived during the wars of the 1990s and 2000s.

After the country houses, as stated, there are areas of rubble currently being occupied by young Venezuelan immigrant couples who, in real time, are building and solving with local means the problems of connectivity to services, such as water, electricity, and television. Next we will discuss these components and how these spaces of habitability are generated that are made outside the optics of planning.

### 2.7 Electricity

The electricity system in the Colombian Caribbean has been characterized as inefficient and plagued by irregularities of all kinds. From a certain point of view, we could say that its formal electricity system is intermittent. The main reason is that the system was appropriated by political elites who, in exchange for bribes, allowed the exploitation of the electrical infrastructure by economic conglomerates that did not make serious investments in or maintain the electricity system. These corporations, the last of them of Spanish origin, fraudulently won the bids to operate the infrastructure, after which they put managers in charge of exploiting the networks without investments, maintenance, or plans to move to environmentally friendly energy sources.

The company that offers electrical energy is called Electricaribe. It is has been taken over by a public authority because it was plundered by a Spanish corporation so that its network became inefficient and kept collapsing. The intervention model is the same: public resources are used to finance the normalization plans, while the lost capital vanishes in an endless legal fight that usually ends up in conciliation centers in Paris or London.

Fortunately, during the pandemic, which officially began on March 16, 2020 in Colombia, the Santa Marta network has worked because it has not had to provide power to the large resorts in Santa Marta. On an ethnographic level, it is clear that, in the Colombian Caribbean, the formal power grid is not 100% reliable; given that we have lived in Taganga for three years, we calculated the cut-off times in 2019 and found that there is only a 75–80% supply per year. The months with the most cuts are July–August and December–January, as the network is overloaded because the area's hotel capacity is at 100%.

The energy supply in the Colombian Caribbean has always been politicized. Thus, since the energy supply network was first configured, the priority was not the communities but the coal mines in the Department of La Guajira, one called El Cerrejón. As with the sale of the electricity system to corrupt corporations, La Guajira's coal rights were given to a U.S. multinational. Its contract, which has always been questioned, assumes that the company is responsible for reporting its extraction at the mine's mouth. On that basis it pays royalties, generating wealth at the local inhabitants' expense. In addition, the Colombian state must guarantee the company's electricity supply to ensure the entire operation.

Along with the energy sold to El Cerrejón below the retail price, the Colombian state must guarantee fuel supplies, primarily diesel and gasoline. During Hugo Chávez's life, fuel was supplied by Venezuelan suppliers; after former president Álvaro Uribe started a dispute with Chávez, companies based on Colombian soil were put in charge of providing the fuel. The Colombian–Venezuelan fight effectively favored the gasoline vendors based in Colombia, which are U.S. companies.

The geopolitical dispute between Colombia and Venezuela, a localized proxy for the disputes of the China–Russia blocs and U.S.–E.U. bloc, generated a local impact both in the Maracaibo region in Venezuela and in La Guajira and Magdalena in Colombia. Since both countries’ families are irrigated between these two nations, the dispute involved a process of border consolidation that prevented the traditional exchanges of food and merchandises. Among them, one that was made difficult was the transfer of Venezuelan gasoline to Colombia. Ten years ago, it was possible to find gasoline sold in Venezuela at a third of the price it sold for in Colombia; it was excellent gasoline with proper combustion that made it desirable. This allowed the popular classes to supply fuel and maintain their motorcycles, cars, and electricity generators. With the crisis between the two countries, Venezuelan gasoline ran out in Colombia, and the lower classes must now pay domestic prices.

As the need to generate settlements in areas such as the Taganga dry forest has arisen, however, families have appealed to local strategies of informal electricity connections. These connections consist of a connection to the proper wiring, a cable to take it to the settlement, and a link, generally at 110 V (see Figure 5).

In the case of electricity, the last formal connections provide power to the country houses; after this area, there is the rubble area, where new settlements using informal links are now beginning to appear. Since several families hang their wires informally on the grid, the network is always overloaded, and daily power outages range from a few minutes to several hours, although outages of more than four hours are rare. In any case, people with more purchasing power have managed to have gasoline or diesel-powered electricity generators that turn on automatically once the normal flow of electricity is gone.

During the pandemic, there was an 18-hour cut. With temperatures ranging from 35–40 °C, the lack of air conditioning is nearly lethal. In addition, during these periods without electricity, students’ work stops and food cold chains are interrupted, so it is safest to buy food every 4 or 5 days; the protein that is best preserved is fish because it only lasts a short time before it is consumed.

### 2.8 Waste

It should be noted that, between the end of the country houses and the beginning of the dump, there is an area where people usually deposit their waste, especially food wrappers. Since the country houses have their own garbage dumps, and since these properties pay the energy company for waste service, it is these spaces the garbage truck passes. Through legal actions we took against the local garbage company, we were able to get management to send a crew to collect the garbage that had been accumulating on our streets. While this should not matter to those living in the cottages, the constant dumping of waste by passersby, usually the inhabitants of the newly built settlements, was untenable. Since the garbage truck passes by on Mondays, Wednesdays, and Fridays from 6:00 AM to 7:00 AM, young waste pickers are looking for beer or soda cans, which are the most valuable. When taking out the garbage, the bags are broken because the recyclers have cut them to look for items they can resell. Every Monday, one of us try to take out the beer bottles and beer cans separately so that the young people do not have to break the bags. According to the recyclers, hardly anyone separates the garbage, so they must rummage through the waste; many times, they come across biological waste, and during the pandemic they now live with the fear that a bottle or a can is contaminated with COVID-19. For these garbage collectors, there are no other options: they must go out to work and risk that they may become infected, and that if they become infected and require medical help, they may not get it because ambulances are only available through private medical services.

Many people living in the new settlements have no possibility of going to supermarkets to buy basic necessities, such as cooking oil, rice, or sugar, for example. So, in the area, some pantries sell these products in smaller quantities, such as 100 mL of cooking oil or 250 g of rice. Drinking water is sold from 500 mL to 5000 mL and packaged in small plastic bags. This minimalist economy generates a significant amount of plastic that ends up in the streets and then in the sea. When people take the trouble to dispose of the waste in bags, they leave it in certain spots where dogs and cats go to look for food. Even before COVID-19, dogs and cats fed on the debris left by tourists, and today there are large populations of hungry cats and dogs, and some local people buy food to appease these feline and canine communities. In the last few years, animal-defense organizations have proliferated, carrying out campaigns to sterilize these animals. We see, then, that pauperization is an issue that affects not only human populations.

### 2.9 Food

Related to food is subsistence if we examine it from an anthropological perspective. If we focus on today's immigrant populations, before the COVID-19 pandemic, most survived by selling snacks to tourists on the beach. This generated a saturation of informal vendors providing various products, such as water, beer, sweets, gum, and empanadas, for example. After the pandemic and with the ban on movement in the beach areas, this population broke up. Some managed to return to Venezuela before the measures were tightened, and others risked their lives to stay. As mentioned by one of the people I managed to talk to:

For us, it is not convenient to go back; we have nowhere to go, our families have no way of receiving us. Here, you know, we defend ourselves. Now we are helping to repair some houses, and we are helping to build others; some of our brothers are going out with the fishermen, and here we have these walls and this roof. (Interview, April 5, 2020).

It is clear that Carlos (a fictitious name) is one of the immigrants who had to settle in the dry forest with his partner. Today she is pregnant, and as I write these lines, Carlos's friend Juan (also a fictitious name) is clearing away rubble to build his walls and his house. They do not perform certified or skilled work, as is the case with other Venezuelan immigrants who have come to replace various car mechanics, carpenters, bricklayers, and plumbers in northern Colombia. These people, however, who have managed to become a little better off, prefer to rent apartments rather than embark on the adventure of building in rubble-filled areas close to a critical national natural park.

Currently, people like Carlos and Juan are working as construction workers, at a time when the Colombian government has allowed some work to reopen; some businessmen have had cash flow and have hired these immigrants to make minor repairs. Moreover, they have money to buy food and support their families. It is clear that these populations have not received help from the Colombian government because they are not in the national social security system's databases. In the pandemic months, this system has provided individual grants of almost $40, but these resources have not reached the immigrant population. As Carlos makes clear, it is better to be outside the system because, otherwise, they would ask where they were living, and some authorities may prevent them from continuing to build their settlements.

What is a fact is that these new settlements are near the sea, so the protein resources are abundant, and the Venezuelan immigrants have connected to the nets of fishers; this means that energy must be invested in earning between $5 and $7 in order to be able to buy carbohydrates and calories, like wheat flour and sugar. Likewise, the owners of the pantries have micro-credit systems so that there are minimum conditions to survive.

### 2.10 Education

As we have observed throughout these two years, the right-wing government of Colombian President Ivan Duque attempted to discredit his Venezuelan counterpart, Nicolas Maduro, who facilitated Venezuelans’ immigration to Colombia. In this way, some young people managed to enter schools, such as Taganga or even the University of Magdalena, but this was by no means true for the majority of the population. In fact, from what is shown in the press, and especially by local activists, the children of Venezuelan immigrants lack food, shelter, and education. The pandemic situation aggravated this condition because these populations were forbidden from loitering in the streets, which were where they obtained resources through begging. In Taganga, education only covers the primary and secondary levels and only covers the local population. Thus, immigrants, who before the pandemic were excluded from the education systems, were now in the pandemic even more so because in the areas where they are building their settlements, first, there is a lack of connectivity, and second, they lack devices like PCs or smartphones to connect to virtual classrooms.

At the university level, the Universidad del Magdalena rector has pointed out that surveys showed that about 30% of the university's 16,000 students were likely to withdraw because they would not be willing to learn via virtual modules. One way to prevent 4,800 people from dropping out was to propose a zero-tuition fee, which has been discussed nationally. In this sense, what COVID-19 has done is to strip students of this vulnerability, in terms of access to internet networks to replace the face-to-face courses that will now have to be virtual until the pandemic ends; likewise, given that many young people have left their impoverished homes for university campuses, confinement has reduced their access to spaces for their free intellectual development.

### 2.11 Water

**Figure 6:**
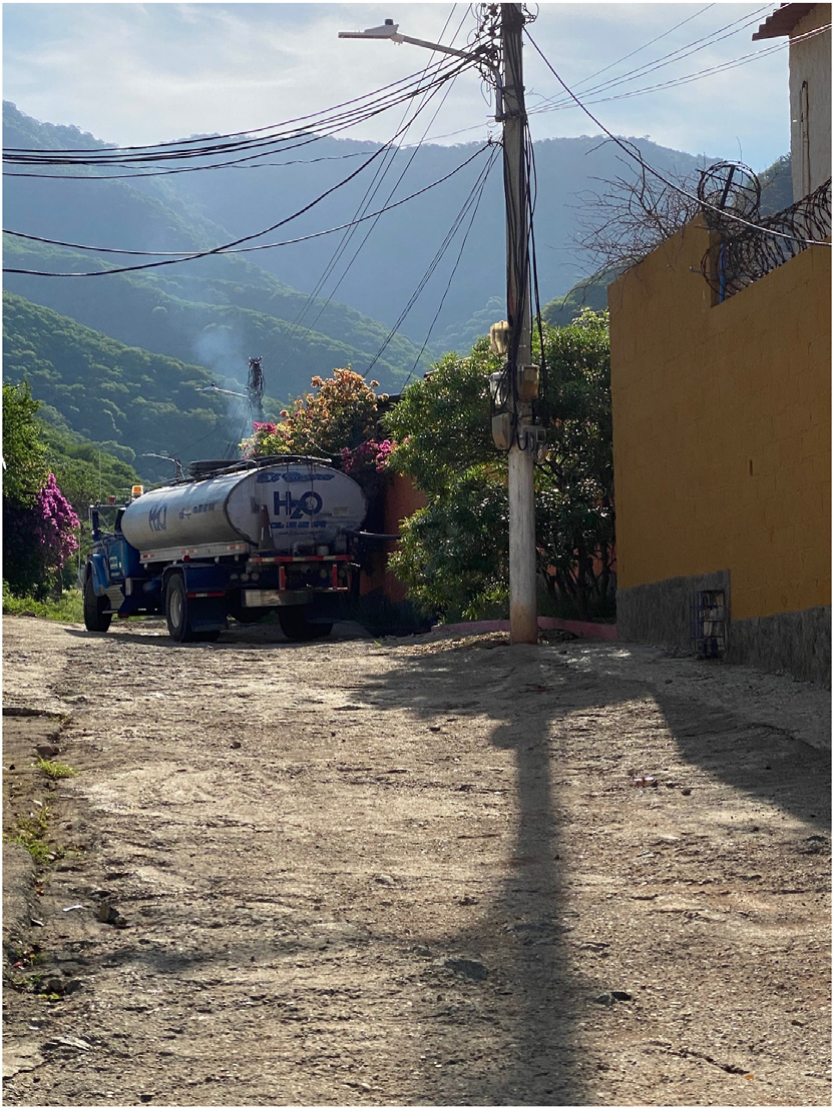
Tank water truck.

Since the dry forest has no running water, the water the ecosystem gets comes from humidity. When it rains, however, small streams form that carry sediment to the sea. At present, the water that arrives at Taganga is brought from wells that draw groundwater. With motor pumps, tank trucks are loaded to store between 9,000 and 12,000 L (see Figure 6).

**Figure 7:**
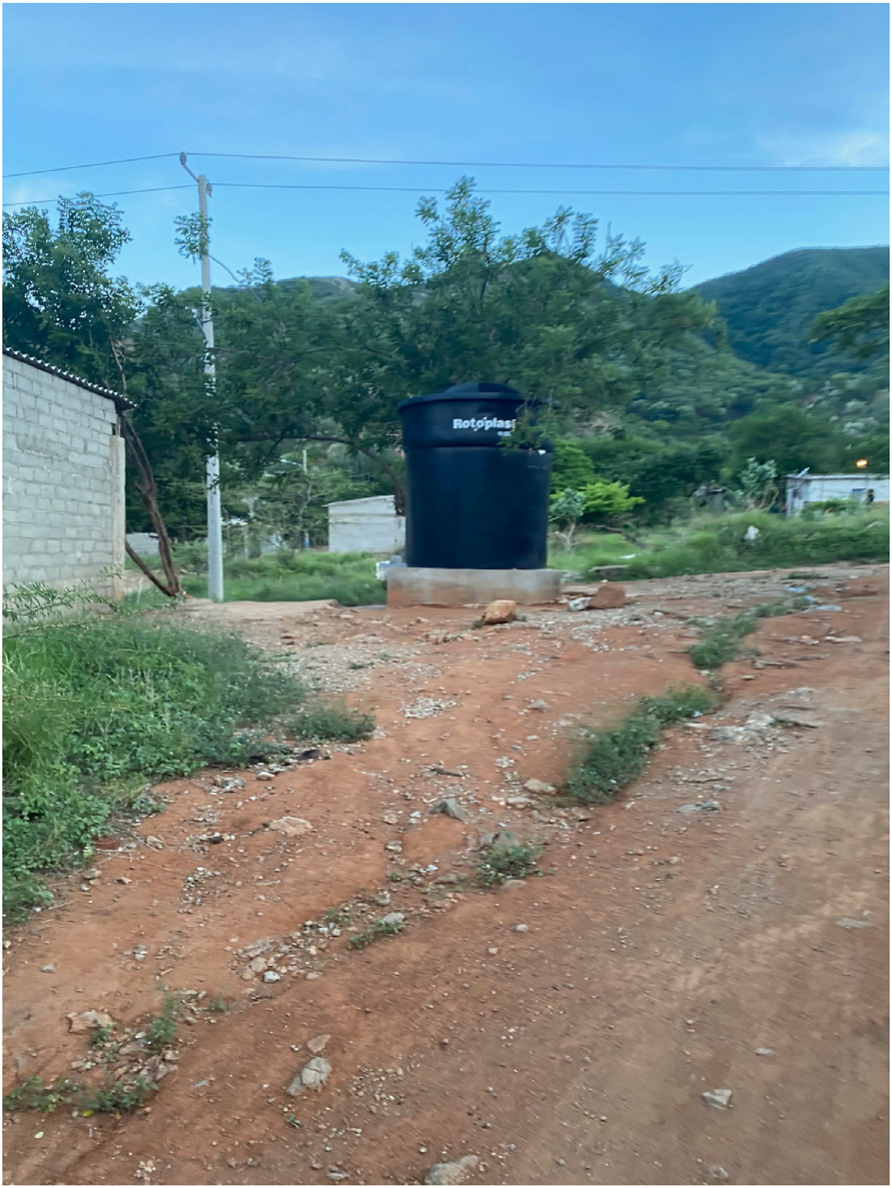
Water distribution tank.

**Figure 8:**
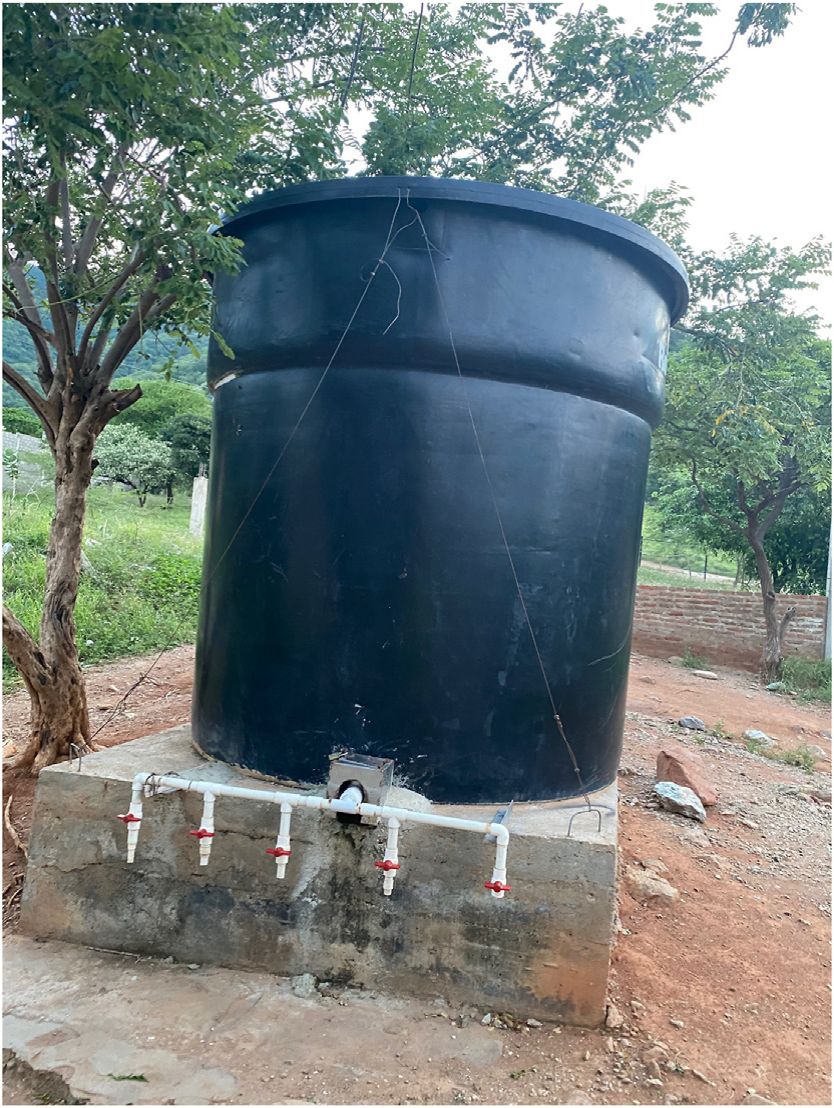
Front view of water distribution tank.

These trucks run on diesel and usually supply the cottages, which consume 12,000 L per week, as a large part of the supply is used to maintain the gardens. We bought half of a tank truck for our own house, which is almost 4,000 L and lasts 20 days. This is worth $25, so the cost of water should be at least $30 a month. Since the immigrant families do not have water, the local government built a pedestal, where they placed a 10,000-liter tank that is filled every week (see Figures 7 and 8).

**Figure 9:**
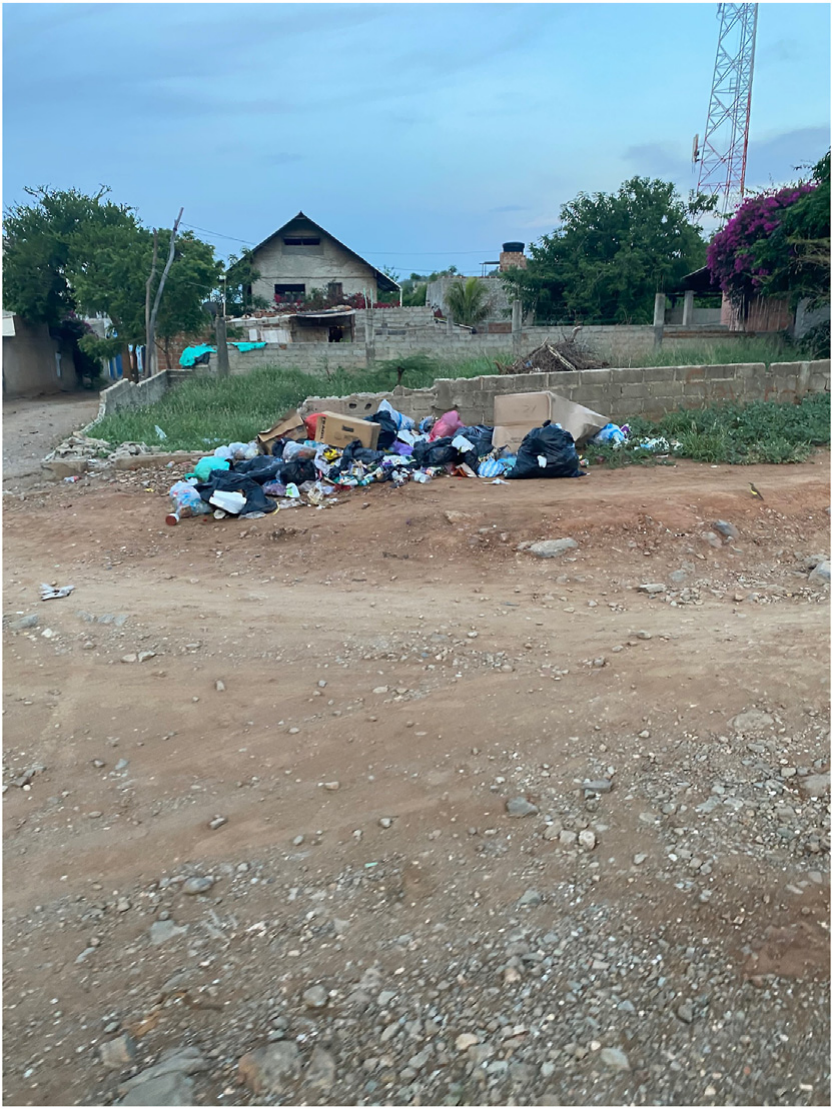
Corner with garbage.

Figure 7 shows the tank in the area where the residents dump their waste for the garbage truck to take away (see Figure 9). Two or three drums (*pimpinas*) per family per day are allowed, but if someone needs more, they may take a little extra. This water is used for household or kitchen purposes and is not used for washing motorcycles or for construction work. As one user pointed out:

The tank is filled once a week; it is always kept full because if there were no water here, people would take the road and not allow cars to enter Taganga. The same thing happens with the electricity: they cannot take it away, or they cannot come and remove these wires because we go out to take the road. If not, let them come and organize the electricity for us, but let them come and offer us jobs; we have nothing to teach. (Interview, July 16, 2020)

## 3 Discussion

Doing ethnography in the global South means facing complex realities. In some cases, the fieldwork is being done in the middle of political and social situations, like Venezuelans’ immigration to Colombia. Unlike the United States or Europe, social stratification in Latin America is not necessarily spatialized, so it is possible to distinguish where the privileged and less privileged live. Like in Taganga, we see extremes of poverty and wealth in the same area, united in the same space. These phenomena have been reported in other parts of the world and are summarized as the existence of precarious spaces where people have to live. In fact, an important part of contemporary anthropological research has to do with recording precarious forms of human existence, as reflected in hundreds of existing slums in large cities, and the ways to transform them into spaces worthy of life (Appadurai, 2000).

These days, it is possible to perceive a process through which large corporations have given some privileges to local elites to keep our countries as supplier of commodities. In this way, as immigration increased, first due to internal violence, and later due to the Venezuelan crisis, the state became a mere administrator of the territories that were important for the enclave economies (Langan, 2017). A corporatist state then emerged that had nothing to do with thinking about social welfare issues, poverty alleviation, or the generation of life opportunities. Most citizens are on their own, and, as we can see, most of them want to build the kind of traditional neighborhoods that can try to do what is traditionally a state responsibility. In this way, as the state functions more as a device to enable the perfect functioning of increase in corporate wealth, as poverty spreads around the globe. The consequences are disastrous, especially as seen in the direct correlation between poverty conditions and child abuse (Atwood, 2003).

In the case of northern Colombia, the state is found in the tolls on the roads, it is found in the military detachments that guard the ports where coal is exported to the United States, but it is not seen in other infrastructure, such as universities, schools, or hospitals. In fact, the real estate sector growing the most is that of hotels and apartment buildings, so the infrastructure for civilian real estate is conspicuous in its absence. For this reason, a fracture is generated in which various authors distinguish, on the one hand, society, and on the other, the state, as two antagonistic forces that are not necessarily complementary (Clastres, 2020).

Although we do not have data that allow us to understand how Santa Marta has grown in the last 30 years, it is clear that urban growth has occurred through settlements that have been planned by immigrants, most of which are later legalized through the granting of titles, and through the setting up of meters for the collection of the kilowatts consumed. Nevertheless, there has not been a clear policy of land management for the welfare of its citizens. We have witnessed a state imprisoned by the desires and whims of transnational corporations, which demand that the state regulate a package of soft regulations so that they can settle in the territory, exploit it, and remove its capital. Thus, the construction of citizenship is in the hands of society without the participation of the state. For this reason, in many parts of Latin America, the construction of neighborhoods, with their spaces for the development of life in the community, is the responsibility of immigrants, without any participation by the state (Sassone, 2007).

Although this is the trend, the residents of Santa Marta and the local inhabitants try to establish their settlements as closely as possible to the full citizenship model, where citizens enjoy various services, such as energy, electricity, and the internet. They have achieved a motivation for life that has been little explored or highlighted in ethnographic studies. As Carla, Carlos's wife, relates:

We are not thieves; we are Venezuelans who had to come because things are not good there. But we do not want anything different than to be able to live in peace, and here we do not do anything wrong, even though people look at us with fear as if we were doing something wrong. We just want to raise a family and for the children to grow up in peace. (Interview, July 19, 2020)

As various studies have shown (Schwartz et al., 2018), the immigration of Venezuelans to Colombia and other parts of the world generates a psychological effect on people, so this attempt to build a neighborhood is a way to mitigate the uncertainty generated by their change of country.

With these elements, then we can answer the questions with which we started the text. There is a social stratification in Colombia of citizens who are first-class and for whom, during the pandemic, intensive care units (ICUs) have been reserved. Then come the Colombian citizens who have no medical insurance and are at the mercy of the national health system, and finally, the Venezuelan immigrants who have no chance of being treated. These citizens are making their settlements to take refuge from the growing wave of contagion under the current conditions. Among them there is no fear of being infected because the real danger is to go back to sleeping on the streets, which would now be lethal for those like Carla, who was pregnant. Our ultimate conclusion is that Venezuelan immigration to neighboring areas, such as Brazil and Colombia, is generating a public health crisis (Doocy et al., 2019; Luna, 2018).

Given this situation, it is clear that there is no plan by the Colombian state to formalize the settlements like the one currently being built in Taganga on the slopes of its hills, and it is also clear that there is a desire to organize these settlements so that newly arrived couples can see their children grow up. The unfortunate aspect of this is that, given the current circumstances, they will have to make their settlements by accessing informal connections and intermittent water supplies. The future seems uncertain, and in these contexts, the demands are only too great for survival. It is clear that the world is going through a crisis in relation to the massive existence of immigrants, refugees, and homeless people (Matlin et al., 2018); This is not an anomaly, but the inevitable result of the consolidation of a global social model.

In a context where it is the citizens who must manage the city itself, the question of the legitimacy of the state emerges. If it is impossible for the state, as in northern Colombia, to offer essential services, such as water, energy, and internet access, what is the state for unless it is to protect foreign conglomerates’ private property? We are facing a crisis of the state because the pandemic has revealed that the state is an institution that governs private capital, leaving the project of building citizenship outside its management. This is a clear example that shows us that the state has collapsed, disappeared, or is an archaeological vestige, although some academics have argued that the Colombian state is made on a day-to-day basis (Ballvé, 2012) and others have argued that it has collapsed (Bejarano and Pizarro, 2004).

Today, Venezuelan immigrants are crowded into the area where they make their settlements; they are generating their reterritorialization dynamics through the use of their music and religious beliefs. So we see that the poverty lines will get starker in the future—not a good sign in an era where the state's capacity to generate welfare has been notably reduced. As this qualitative research shows, in immigrant host territories, such as the north of Colombia, a chaotic image can be perceived in which the increase in population runs parallel to an increasingly weak state in terms of generating well-being for its citizens.

The future will, then, mean thinking more about models based on local food sovereignty management and less about territories as spaces that offer raw materials. The model imposed by the United States throughout the configuration of Latin America as a supplier of commodities has reached its limits, and it is necessary to think of terms of forms of local management that guarantee the well-being of the residents of these territories (Escobar, 2011).

From an analytical point of view, we can appreciate that contemporary immigration, such as that of Venezuelans to Colombia, occurs within a structural conflict of global scope, such as the dispute over Venezuelan hydrocarbons. The depreciation of the Venezuelan currency, plus the economic blockade, added to internal corruption, have made the alternative the pursuit of better opportunities in tourism areas, such as Colombia. The data also show that these contemporary inhabitants of the Taganga dry forest do not have formal schooling, so it is clear that immigration to tourist areas is being done by people without any academic qualifications.

These determinants suppose a perpetuation of poverty conditions, since the children of these immigrants, born on Colombian soil, will lack access to education, which will diminish their lives’ horizons, reducing them to mere survival and the consumption of television and social networks.

A strong recommendation that emerges from this study, and that can be welcomed by planners and policy makers, is that, in the face of the public health crisis that is being structured, it is necessary to make visible immigrants’ struggles to build public spaces and neighborhoods. When demonstrating these practices, one must consider how the state recovers its function as an instrument of social development and not just as a device for the reproduction of capital. Without a doubt, policy makers should structure policies that start from principles outside the neoliberal logic, in such a way that the priority is the organization of the occupation by immigrants, from a human and ethical point of view.

## Declarations

### Author contribution statement

Wilhelm Londoño Díaz: Conceived and designed the experiments; Performed the experiments; Analyzed and interpreted the data; Contributed reagents, materials, analysis tools or data.

Anghie Prado Mejía: Analyzed and interpreted the data; Wrote the paper.

### Funding statement

This work was supported by the 10.13039/501100016363University of Magdalena.

### Data availability statement

Data included in article.

### Declaration of interests statement

The authors declare the following conflict of interests.

### Additional information

No additional information is available for this paper.
